# Possible roles of deep cortical neurons and oligodendrocytes in the neural basis of human sociality

**DOI:** 10.1007/s12565-023-00747-1

**Published:** 2023-11-27

**Authors:** Noriyoshi Usui

**Affiliations:** 1 Department of Neuroscience and Cell Biology, Graduate School of Medicine, Osaka University, Suita, 565-0871 Japan; 2 Omics Center, Center of Medical Innovation and Translational Research, Graduate School of Medicine, Osaka University, Suita, 565-0871 Japan; 3 United Graduate School of Child Development, Osaka University, Suita, 565-0871 Japan; 4 Global Center for Medical Engineering and Informatics, Osaka University, Suita, 565-0871 Japan; 5 Addiction Research Unit, Osaka Psychiatric Research Center, Osaka Psychiatric Medical Center, Osaka, 541-8567 Japan

**Keywords:** Sociality, Brain evolution, Autism spectrum disorder, Deep layer, Oligodendrocyte

## Abstract

Sociality is an instinctive property of organisms that live in relation to others and is a complex characteristic of higher order brain functions. However, the evolution of the human brain to acquire higher order brain functions, such as sociality, and the neural basis for executing these functions and their control mechanisms are largely unknown. Several studies have attempted to evaluate how human sociality was acquired during the course of evolution and the mechanisms controlling sociality from a neurodevelopment viewpoint. This review discusses these findings in the context of human brain evolution and the pathophysiology of autism spectrum disorder (ASD). Comparative genomic studies of postmortem primate brains have demonstrated human-specific regulatory mechanisms underlying higher order brain functions, providing evidence for the contribution of oligodendrocytes to human brain function. Functional analyses of the causative genes of ASD in animal models have demonstrated that the neural basis of social behavior is associated with layer 6 (L6) of the neocortex and oligodendrocytes. These findings demonstrate that both neurons and oligodendrocytes contribute to the neural basis and molecular mechanisms underlying human brain evolution and social functioning. This review provides novel insights into sociability and the corresponding neural bases of brain disorders and evolution.

## Introduction

**Fig. 1:**
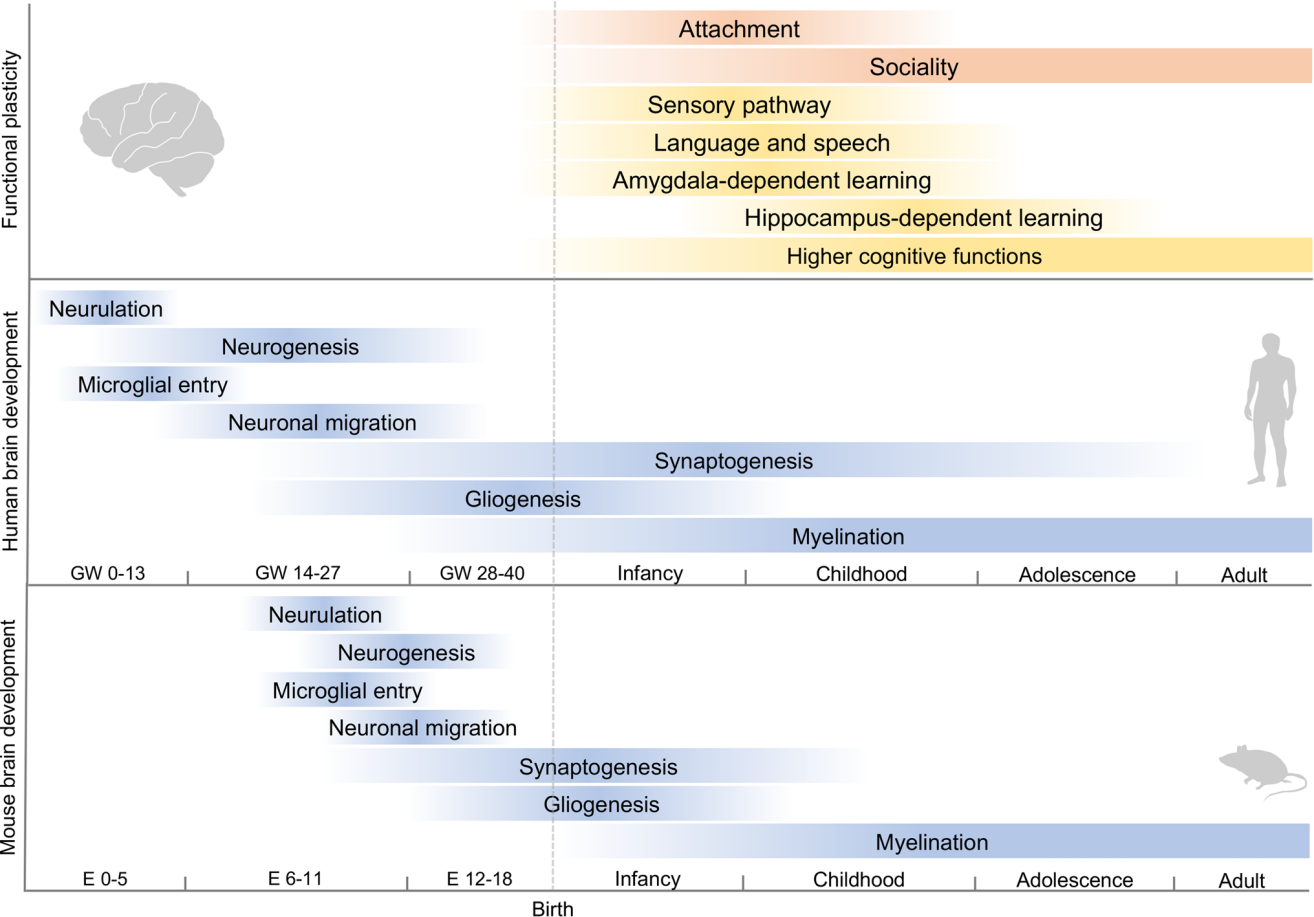
Developmental trajectories of the brain and plasticity. The pink bars at the top indicate social plasticity. The attachment was the first social bond formed with caregiver. Sociality develops through interactions with others in a social environment. Middle yellow bars indicate brain functional plasticity events. The sensory pathway includes functions associated with the visual, auditory, and somatosensory cortices such as vision, hearing, and touch, respectively. Language and speech include functions associated with the angular gyrus and Broca’s area. Amygdala-dependent learning includes cued conditioning and implicit learning. Hippocampus-dependent learning involves learning objects, places, spaces, and work. Higher cognitive function is associated with the prefrontal cortex (PFC). The bottom blue bars indicate developmental brain events in human and mouse. After neural tube formation in the ectoderm (neurulation), brain vesicles are formed, and neuroepithelial cells produce neural stem cell progenitors and neurons (neurogenesis). Neural progenitor cells also produce glia, such as astrocytes and oligodendrocytes (gliogenesis). Developing neurons migrate, differentiate into specific subtypes, form synapses, and become myelinated. *GW* gestational week

Sociality is a higher order brain function that is essential for living within a community. Sociality is formed through relationships with others, and accumulating social experience leads to higher levels of sociality (Kappeler et al. 2015; Sachser et al. 2013; Tomasello 2020). Sociability begins with the formation of attachment to the caregiver after birth, and advanced sociability is acquired during different stages of growth from childhood (Tomasello 2020; Cascio et al. 2019; Jethava et al. 2022; Vivanti and Nuske 2017). Impairment in attachment formation during childhood negatively affects children’s social development (Teicher et al. 2016). Sociality and brain development are closely related (Fig. 1), and brain developmental disorders during the prenatal period increase the risk of postnatal neurodevelopmental and psychiatric disorders (Usui et al. 2023; Li et al. 2023; Doi et al. 2022a, b).

Previous studies demonstrated that several brain regions are associated with sociality. However, sociality is related to various factors, such as emotion, anxiety, and cognition, and cannot be explained by a specific circuit because of its complexity. Clinical studies on autism spectrum disorder (ASD), a neurodevelopmental disorder (NDD), have identified various brain regions associated with social interactions and communication (Amaral et al. 2008). For example, the orbitofrontal cortex, anterior cingulate cortex, and amygdala mirror neuron regions have been reported to be responsible for social interaction, while the inferior frontal gyrus, superior temporal sulcus, and basal ganglia are responsible for communication (Amaral et al. 2008; Hadjikhani et al. 2006). Moreover, studies using animals with modifications in genes responsible for ASD have reported developmental abnormalities in various brain regions involved in social behavior (de la Torre-Ubieta et al. 2016; Wang et al. 2014; Tebbenkamp et al. 2014; Doi et al. 2022a, b; Quesnel-Vallières et al. 2019; Willsey et al. 2022). Many genes related to human genetic risks associated with brain development and disorders, such as ASD and schizophrenia (SCZ), have been identified to understand the molecular mechanisms underlying social behavior (Doi et al. 2022a, b; Doan et al. 2018).

Humans have built a more advanced civilization than other primates. In this process, various events such as biological evolution, the evolution of the living environment and civilization, and population increases have led to the acquisition of high-level and complex functions (Russo and Nestler 2013; Usui et al. 2014). Previous studies on primates reported a positive correlation between the size of social groups and the neocortex capacity (Silk 2007; Dunbar and Shultz 2007). Language has evolved as a tool for social communication during the course of evolution (Russo and Nestler 2013; Usui et al. 2014). However, acquiring higher order brain functions is often accompanied by the risk of developing disorders that can impair these functions, such as NDDs, psychiatric disorders, and cognitive impairment (Usui et al. 2014; Irie et al. 2022; Pattabiraman et al. 2020; Kappeler et al. 2015; Vanderhaeghen and Polleux 2023).

Previous studies show that the acquisition and development of sociality could be closely related to brain development and influenced by the individual’s genes and the growing environment (Fig. 1). This review discusses the neural basis underlying sociality from the relevant studies of human brain evolution and disorders that impair sociality such as ASD and SCZ. In addition, I will also discuss the prospects of research aimed at elucidating neural circuits related to sociality, and research aimed at elucidating the functions of human-specific gene control mechanisms in the acquisition of higher brain functions in humans.

## Human-specific genes and their functions in human brain evolution

The human brain has the largest proportion of body size among mammals. Various findings have been reported in the evolution of the human brain. Human neurons have larger cell bodies, thicker dendrites, and axons, with increased numbers and density of spines in pyramidal cells (Defelipe 2011; Elston et al. 2001; Duan et al. 2003). A recent connectome study comparing the cerebral cortices of humans, macaque monkeys, and mice reported that humans have a threefold increase in the number of interneurons and tenfold expansion of the interneuron-to-interneuron network compared with mice (Loomba et al. 2022).

Human-specific genes and their regulation machinery helped evolve the modern human brain (Usui et al. 2014; Irie et al. 2022; Florio et al. 2017). *SRGAP2* gene has been duplicated thrice during human evolution, subsequently evolving as *SRGAP2B, SRGAP2C, and SRGAP2D* (Geschwind and Konopka 2012; Charrier et al. 2012; Dennis et al. 2012). Human-specific *SRGAP2C* increases spine density and delays maturation in the neocortex (Geschwind and Konopka 2012; Charrier et al. 2012), suggesting that *SRGAP2C* contributes to cortical expansion and increased spine numbers in humans. *ARHGAP11B* arose from the duplication of *ARHGAP11A* in humans and is expressed in the human apical and basal radial glia (Florio et al. 2015). *ARHGAP11B* promotes the generation and self-renewal of basal progenitors in the mouse cortex and increases cortical size and folding in the neocortex of marmosets (Florio et al. 2015; Heide et al. 2020). *ARHGAP11B* also regulates the mitochondrial Ca^2+^ concentration and induces glutaminolysis, which is required for human basal progenitor proliferation (Namba et al. 2020).

In addition, *NOTCH2NL*, human-specific paralogs of the NOTCH2 receptor, plays a role in the expansion of the human cortex by promoting cortical progenitor maintenance to generate higher neuronal outputs through inhibition of cis Delta/Notch interactions (Suzuki et al. 2018). Another group also reported that different alleles of *NOTCH2NL* enhance Notch signaling by directly interacting with NOTCH receptors to maintain the proliferation (Fiddes et al. 2018). In addition, *NOTCH2NL* located in 1q21.1 where distal deletions are associated with microcephaly and schizophrenia, and duplications are associated with macrocephaly and ASD, respectively (Fiddes et al. 2018). Hominini-specific regulation of *CBLN2* having species differences in level of expression and laminar distribution promotes PFC dendritic spine formation (Shibata et al. 2021). *CROCCP2* expressed in human fetal cortex also promotes proliferation of cortical progenitors by acting as a human-specific modifier to cilia dynamics and mTOR signaling (Van Heurck et al. 2023). Primate-specific gene *TMEM14B* expressed in outer radial glia cells also plays a role in cortical expansion and folding by interaction with IQGAP1 (Liu et al. 2017).

Moreover, the molecular evolution of *FOXP2* identified from the KE family associated with language disorders is important for human signatures (Lai et al. 2001). Human FOXP2 has two amino acid mutations, T303N and N325S, compared to chimpanzees (Enard et al. 2002; Konopka et al. 2009), which induce functional changes in the FOXP2 transcription factor, resulting in changes in the motor and craniofacial development necessary for language and speech functions (Konopka et al. 2009), as well as diversity in vocalization (Enard et al. 2009).

Furthermore, we demonstrated that characteristic human gene expression networks are important for human brain evolution and brain disorders. A comparative genomic study among primates has shown that *CLOCK* is specifically upregulated in the human prefrontal cortex (PFC) and has identified it as a hub gene involved in cognitive function rather than in controlling circadian rhythms (Konopka et al. 2012). We found that human *CLOCK* regulates gene expression networks involved in cognitive functions, ASD, intellectual disability, as well as neurodevelopment, such as neuronal migration (Fontenot et al. 2017).

**Fig. 2:**
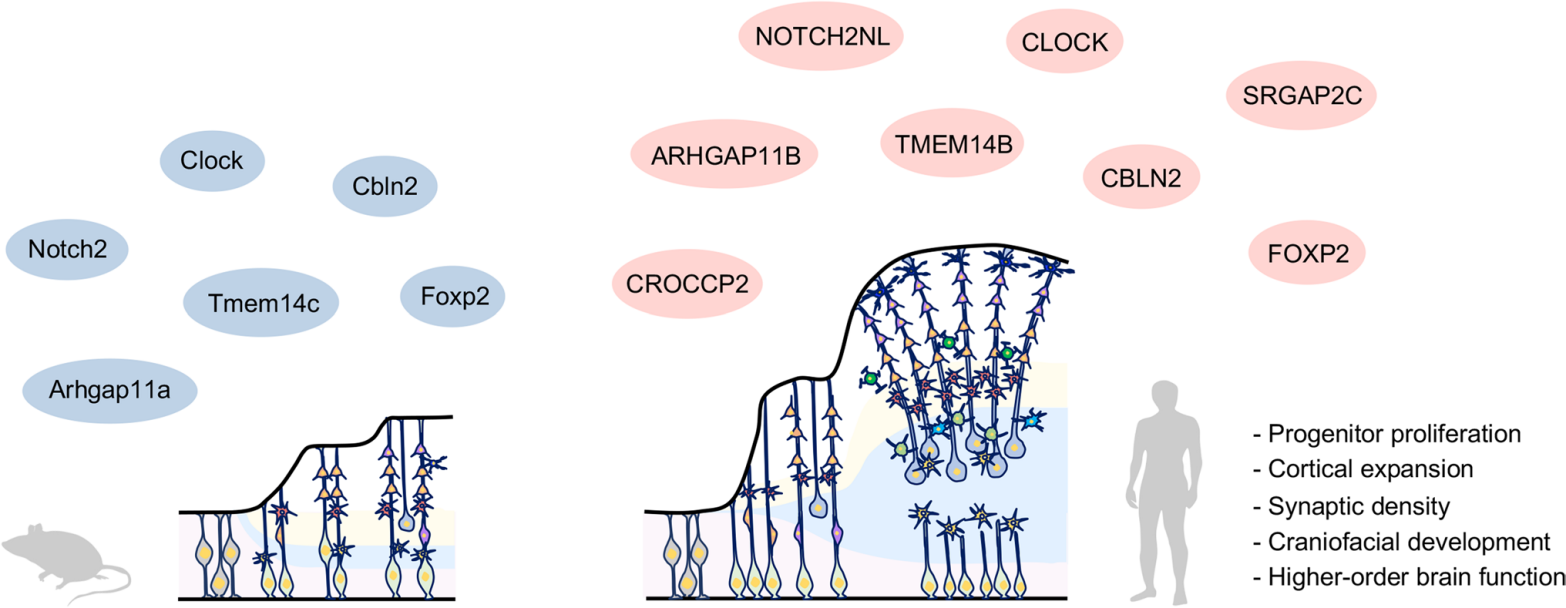
Schematic diagram of human-specific genes and its functions in human brain evolution. The blue color and pink color indicate the mouse genes and human genes, respectively. Human-specific genes accelerate evolution and expansion of human brain and its functions. These genes contribute to progenitor proliferation, expansion, increased numbers of synapses, and acquisition of higher order brain functions in human brain. Briefly, *ARHGAP11B* regulates basal progenitor proliferation. *CROCCP2* promotes cortical progenitor proliferation. *NOTCH2NL* promotes cortical progenitor maintenance and cortical expansion. *TMEM14B* promotes cortical expansion. *CLOCK* also regulates neurodevelopmental gene expressions and neuronal migration. *CBLN2* promotes dendritic spine formation. *SRGAP2C* increases spine density. *FOXP2* regulates expression of genes associated with motor and craniofacial development

These findings suggest that human-specific gene functions are involved in the expansion of the human brain and the increase in synapses, contributing to neurodevelopment and acquisition of higher order brain functions (Fig. 2).

## Oligodendrocytes contributions to human brain evolution

It has been argued that the human brain has an increased number of glia compared to neurons. The ratio of neurons to glia is said to increase from 1:1 in mice to 1:10 in humans. However, this signature has not been proven, and several studies have reported negative results regarding the tenfold increase. In fact, it has been reported that the ratio is 1:1 or slightly more glia in humans (Sherwood et al. 2006; Herculano-Houzel 2009, 2012, von Bartheld et al. 2016), but the actual ratio remains unknown because quantification in the whole brain has not been performed.

Glia were once thought to be supportive cells that fill gaps in the brain, but the importance and function of glia in the brain is becoming better understood. Glia in the central nervous system include astrocytes, oligodendrocytes, and microglia. Astrocytes physically support neurons, interact with brain blood vessels, reuptake of glutamate between synapses, and regulate extracellular ion concentrations (Volterra and Meldolesi 2005; Abbott et al. 2006). Oligodendrocytes form myelin sheaths, which increase the conduction velocity of action potentials by saltatory conduction and play a role in the metabolism of neurons and astrocytes (Richardson et al. 2006; de Faria et al. 2021). Microglia control immunity in the central nervous system through phagocytosis, release humoral factors, and performs synaptic pruning and interactions (Butovsky and Weiner 2018; Li and Barres 2018). Single-cell transcriptome studies have revealed an enormous diversity of glia that play a role in brain development (Fan et al. 2020; Darmanis et al. 2015; Polioudakis et al. 2019; Huang et al. 2020). Furthermore, the engraftment of human astrocytes into the mouse brain has been reported to alter synaptic plasticity and improve learning ability in mice (Han et al. 2013). These studies suggest that human neurons and glia contribute more to higher order brain functions than other primates and rodents.

We also conducted comparative genomics on primates and identified human-specific gene expression network modules in both neurons and oligodendrocytes (Berto et al. 2019; Mendizabal et al. 2019). Interestingly, a human-specific oligodendrocyte module was found to be involved in transcription and histone methylation (Berto et al. 2019). In addition, these module genes are enriched in risk variants such as NDDs, psychiatric disorders, and cognition (Berto et al. 2019). These findings suggest that not only neurons but also oligodendrocytes contributed to the acquisition of higher order brain functions in humans during evolution, and oligodendrocyte impairment is also a risk factor for NDDs and cognitive disorders. Thus, oligodendrocytes play essential roles in human brain evolution and brain functions such as sociality and cognition.

Regarding oligodendrocyte development, neural stem cells generate glia progenitor cells, which differentiate into oligodendrocyte progenitor cells (OPCs). OPCs further differentiate into immature, mature, and myelinated oligodendrocytes (Fields 2015). In human, the frontal cortex is still myelinating beyond late adolescence (Miller et al. 2012). Furthermore, complete myelination occurs only at sexual maturity in chimpanzees, whereas in humans, myelination is expanded compared to that of other primates (Miller et al. 2012), suggesting that oligodendrocytes and their myelination play a role in the development of higher order brain functions and cognition. Like neurogenesis, oligodendrogenesis occurs in the adult brain, and dead oligodendrocytes undergo periodic turnover in human (Rivers et al. 2008). Oligodendrocyte production and myelination peak at the age of 5 in humans and decline yearly after that, and the lost oligodendrocytes are replaced by neogenesis (Yeung et al. 2014). Recently, it has been reported that outer radial glial cells produce EGFR-expressing pre-OPCs in the subventricular zone (SVZ) and that pre-OPCs proliferate to increase the number of mitotic OPCs in the SVZ of human (Huang et al. 2020). They also reported that pre-OPCs proliferate and differentiate into early and late OPCs in the SVZ and subplate of human, respectively (Huang et al. 2020).

However, the human-specific functions of oligodendrocytes remain largely unknown. The human brain undergoes white matter volume expansion (Rilling and van den Heuvel 2018; Donahue et al. 2018). We identified human-specific transcriptome signatures of oligodendrocytes in postmortem brains (Berto et al. 2019); thus, analyses of these gene functions and/or networks may uncover human oligodendrocyte function. Interestingly, the human-specific upregulated neuronal module significantly overlapped with genes in the neuronal module dysregulated in SCZ and ASD in data from the PsychENCODE Consortium (Berto et al. 2019; Gandal et al. 2018). In addition, the human-specific downregulated oligodendrocyte module also overlaps with the genes in the oligodendrocyte module dysregulated in SCZ, ASD, and bipolar disorder in data from the PsychENCODE Consortium (Berto et al. 2019; Gandal et al. 2018). Another study also reported that hominin-specific gene regulatory elements such as enhancers and promoters function as transcriptional units selectively emerged in human oligodendrocytes-lineages; however, these elements were disrupted in patients with ASD (Castelijns et al. 2020). These results suggest that human-specific characteristics of gene expression networks and oligodendrocytes contribute to human brain evolution and sociality.

## Development and impairment of sociality

In addition to normal brain development, the postnatal environment is an important factor in the development of sociality (Fig. 1). The environment influences optimal growth and health, including developmental aspects related to the social, cognitive, and immune systems in children (Consiglio and Brodin 2020; Ferguson et al. 2013; Mackes et al. 2020; Sonuga-Barke et al. 2017). The childhood environment significantly impacts brain structure, synaptic plasticity, and mental development (Miguel et al. 2019; Takesian and Hensch 2013). The period immediately after birth through adolescence, which is characterized by the development and acquisition of various senses, is called the critical period (Fig. 1) (Reh et al. 2020; Alberini and Travaglia 2017). The critical period peaks early in life, during which the nervous system is highly plastic and dynamically regulated throughout life (Takesian and Hensch 2013).

Attachment is the first social and emotional bond fostered between the caregiver and the child in human. Studies show that the critical period for attachment formation is up to the postnatal day (P) 16 in mice (Landers and Sullivan 2012), but the actual critical period has not been established. Impaired attachment formation during childhood increases the risk for attachment disorders in human. Attachment disorders are classified into reactive attachment disorders and disinhibited social engagement disorders (American Psychiatric Association 2013). Odors and touch stimuli are thought to be important for attachment formation in animals (Landers and Sullivan 2012; Sakano 2020). Oxytocin, a well-known social and affective hormone, plays an important role in rodent imprinting (Roth et al. 2013). Oxytocin-mediated imprinting has a critical period in mice (Inoue et al. 2021; Sakano 2020), suggesting that maternal odor during the neonatal period is important for attachment formation in mice. However, the neural basis underlying attachment and social formation remains largely unknown. At present, we have successfully generated a mouse model for attachment disorders and are attempting to elucidate the underlying mechanisms of attachment formation and its neural basis by studying the pathogenesis of attachment disorders.

Childhood stress, also called early life stress, broadly refers to stress experienced before reaching adulthood and includes events such as neglect, physical and psychological abuse, sexual abuse, loss of a caregiver, relationship development, bullying, accidents, illnesses, natural disasters, and wars (Heim et al. 2003, 2004; Agid et al. 2000). Environment is a crucial factor in providing optimal growth and health conditions for children, including social, cognitive, and immune system-related aspects (Consiglio and Brodin 2020; Ferguson et al. 2013; Mackes et al. 2020; Sonuga-Barke et al. 2017). A previous study reported that postnatal socially isolated mice exhibited impairments in social interactions, working memory, and myelination in the PFC (Makinodan et al. 2012). We previously reported that longer periods of social isolation reduced social behaviors in mice, increased anxiety-like behavior, and reduced the number of neurons in the PFC of mice (Usui et al. 2021b). These studies suggest that postnatal environment and social experiences are critical for development of sociality.

Based on socially isolated mice studies, we identified *Zbtb16* as a gene involved in sociality. *Zbtb16* is the most significantly downregulated gene in the PFC of socially isolated mice (Usui et al. 2021b). *ZBTB16* encodes a transcription factor that contains a BTB/POZ protein–protein interaction domain at its N-terminus and a C2H2-type zinc finger DNA-binding domain at its C-terminus, which plays key roles in stem cell maintenance, proliferation, differentiation, apoptosis, and chromatin remodeling Suliman et al. 2012; Šeda et al. 2017). Behavioral analysis was performed to investigate whether *Zbtb16* regulates mouse social behaviors, and it was found that social behavior was impaired in *Zbtb16* knockout (KO) mice (Usui et al. 2021a). Interestingly, this mouse had impaired oligodendrocyte development and differentiation and reduced myelination in the neocortex (Usui et al. 2021a). Oligodendrocyte development has also been reported to be impaired when normal development in mice childhood is disrupted by external factors such as early life stress (Kokkosis et al. 2022; Teissier et al. 2020). Taken together, these findings suggest that oligodendrocytes play an important role in social development and form the neural basis underlying sociality.

## Cortical deep layers underlying sociality

To understand the neural basis of sociality, we focused on ASD as a disorder that impairs sociality. ASD is a heterogeneous NDD that causes pervasive abnormalities in social communication, repetitive restricted behaviors and interests, and hyperesthesia and hypesthesia (Lord et al. 2020, 2018; Khodosevich and Sellgren 2023). ASD pathogenesis is associated with complex genetic and environmental factors (Lord et al. 2020, 2018; Doi et al. 2022a, b; Usui et al. 2023, 2022a; Willsey et al. 2022). Over 1000 ASD-associated genes have been identified in individuals with ASD (Wilkinson et al. 2015; Toma et al. 2014; Griswold et al. 2015; Yao et al. 2015; Satterstrom et al. 2020; Wang et al. 2020; Iossifov et al. 2014; Willsey et al. 2022), which play essential roles in fetal brain development, particularly in neurodevelopment and synaptogenesis (de la Torre-Ubieta et al. 2016; Wang et al. 2014; Tebbenkamp et al. 2014; Doi et al. 2022a, b; Quesnel-Vallières et al. 2019; Willsey et al. 2022).

**Fig. 3:**
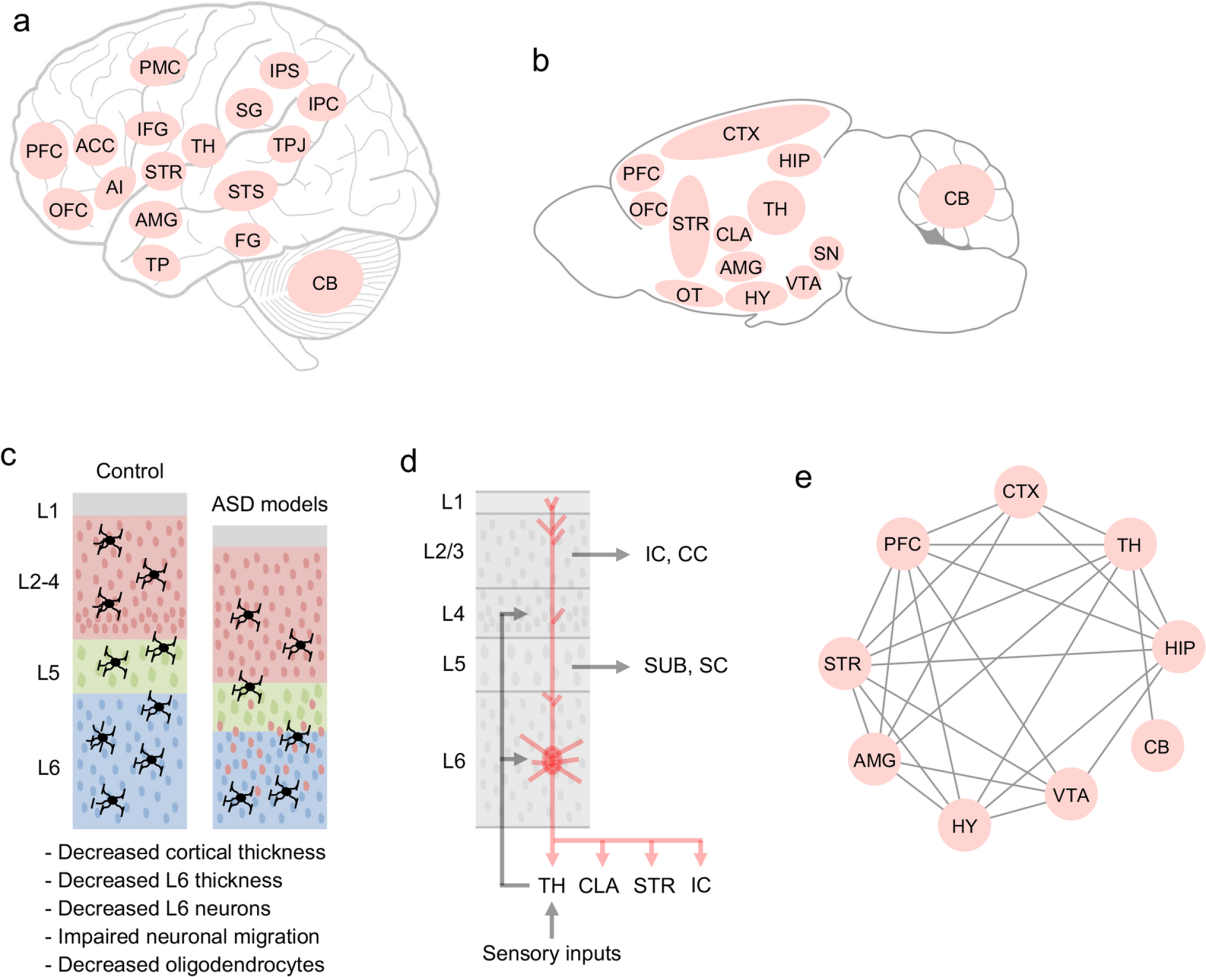
Schematic diagram of human and mouse neural circuits related to social behaviors. a, b Networks of the human (**a**) and mouse (**b**) brain regions involved in sociality. Brain regions associated with sociality have been identified in ASD studies (Amaral et al. 2008; Barak and Feng 2016; Gandhi and Lee 2020). In mice, we identified brain regions associated with sociality, as reported in previous studies, including ours. **c** Schematic diagram of neocortical phenotypes in ASD model mice. From the phenotypes of the multiple ASD model mice, we found decreased cortical thickness, L6 thickness, L6 neurons, impaired neuronal migration, and decreased oligodendrocytes. **d** Schematic diagram of the neural circuits centered at L6 of the neocortex, which are thought to be associated with social behavior. L6 neurons form a feedback loop with the thalamus and play a role in switching behavior by adjusting sensory information, such as visual and auditory. In addition to the thalamus, L6 neurons project to the claustrum, striatum, and ipsilateral cortex, which are associated with social behaviors. **e** Connectome of representative brain regions in mouse social behavior. Abbreviations of each brain region and their roles in social behavior and/or ASD characteristics as follow; *PFC* prefrontal cortex (social information processing), *ACC* anterior cingulate cortex (social cognition), *OFC* orbitofrontal cortex (social adjustment), *TP* temporal pole (language, theory of mind), *FG* fusiform gyrus (face perception), *PMC* premotor cortex (mirror system), *IFG* inferior frontal gyrus (frontal mirror area and emotional judgment), *AI* anterior insula (social cognition), *AMG* amygdala (social information judgment, emotion recognition, and theory of mind), STR striatum (serotonin and dopamine signals), STS superior temporal sulcus (responses to face expression, eye gaze direction, and voice perception), *SG* supramarginal gyrus (mirror system), *TPJ* temporoparietal junction (beliefs and theory of mind), *IPS* interparietal sulcus (eye gaze direction and social status judgment), *IPC* inferior parietal cortex (mirror system), *CTX* cortex, *CLA* claustrum (social behavior, multi-sensory processes, attention, and consciousness), *HIP* hippocampus (social memory and LTP), *TH* thalamus (social recognition and multi-sensory processes), *HY* hypothalamus (oxytocin release, social reward, and stress), *OT* olfactory tubercle (social response, multi-sensory processes, and reward), *VTA* ventral tegmental area (dopamine release and movement), *SN* substantia nigra (dopamine release and movement), *CB* cerebellum (vocal communication), *SC* spinal cord, *L* layer, *IC* ipsilateral cortex, *CC* contralateral cortex, *SUB* subcortical nucleus including the striatum, red nucleus, pontine nucleus, olive nucleus, and spinal cord

Studies in humans have identified various regions in the brain associated with social interactions and communication in individuals with ASD (Amaral et al. 2008). The orbitofrontal cortex, anterior cingulate cortex, and amygdala mirror neuron regions are responsible for social interactions (Fig. 3) (Amaral et al. 2008; Hadjikhani et al. 2006). The inferior frontal gyrus, superior temporal sulcus, and basal ganglia are responsible for communication (Fig. 3) (Amaral et al. 2008). In animal studies, we demonstrated that developmental defects in layer 6 (L6) neurons and oligodendrocytes, which are common in ASD model mice, are responsible for sociality (Fig. 3) (Co et al. 2020a, b; Usui et al. 2017a, 2021a). Studies also demonstrated that Purkinje cells in the cerebellum play a role in social communication (Fig. 3) (Usui et al. 2017b). These findings indicate that brain regions associated with social interaction and communication in humans are consistent with those impaired in mouse models of ASD-causing genes.

*FOXP1* is a forkhead transcription factor that regulates cell proliferation and differentiation during development and is a high-confidence gene associated with ASD (Iossifov et al. 2014; Sanders et al. 2015; Stessman et al. 2017; Satterstrom et al. 2020; Willsey et al. 2022; Bacon and Rappold 2012; Siper et al. 2017; Hamdan et al. 2010; Lozano et al. 2015; O'Roak et al. 2011). Deletion of *Foxp1* in the brain using a conditional knockout (cKO) (*Nes-Cre*; *Foxp1*^*flox/flox*^) has been reported to cause ASD-like behaviors, abnormalities in the striatum and hippocampal development, and reduced excitability of hippocampal CA1 neurons in adult mice (Bacon et al. 2015). Patient-relevant *Foxp1* mouse studies have reported increased excitability of striatal medium spiny neurons, reduced neonatal ultrasonic vocalizations (USVs), and altered gene expression related to ASD in adult mice (Fig. 3) (Araujo et al. 2015). We have also demonstrated that forebrain-specific *Foxp1* cKO (*Emx1-Cre*; *Foxp1*^*flox/flox*^) mice show impaired neonatal and adult USVs, global motor dysfunction, social impairment, hyperactivity, and anxiety-like behavior in postnatal and adult mice (Usui et al. 2017a; Araujo et al. 2017). In *Foxp1* cKO mice, reduced neocortical thickness, particularly L6 thickness, and fewer L6 neurons were observed (Fig. 3) (Usui et al. 2017a). Analysis of *Foxp1*-transcriptome in the PFC and hippocampus revealed that *Foxp1* regulates cell proliferation and differentiation, cell migration, synaptic transmission, axon ensheathment, and ASD-associated gene expression (Araujo et al. 2017; Usui et al. 2017a). These findings demonstrate that the L6, the striatum, and the hippocampus, including electrophysiological properties, are essential for the neural basis of sociality.

*FOXP2* is a member of the forkhead gene family and is expressed in deep-layer subcortical projection neurons, including L6 corticothalamic projection neurons and L5 pyramidal tract neurons (Sorensen et al. 2015; Tasic et al. 2016; Kast et al. 2019; Willsey et al. 2013). Mutations of *FOXP2* have been identified in patients with speech and language disorders, ASD, and attention-deficit/hyperactivity disorder (Co et al. 2020a, b; Lai et al. 2001; Demontis et al. 2019; Reuter et al. 2017; Satterstrom et al. 2020). We have previously shown that Foxp2 regulates vocal communication and motor functions in postnatal mice through the development of cerebellar Purkinje cells (Fig. 3) (Usui et al. 2017b). Moreover, cortex-specific *Foxp2* cKO (*Emx1-Cre*; *Foxp2*^*flox/flox*^) mice showed decreased neonatal USVs, impaired cognitive flexibility and hyperactivity, and decreased cortical dopamine receptor D1 (DRD1) expression (Fig. 3) (Co et al. 2020a, b). These studies demonstrate that the neural basis of cerebellar Purkinje cells and L6 is important for sociality.

*ZBTB16* transcription factor plays a role in cell proliferation and differentiation, apoptosis, chromatin remodeling, and other biological functions (Suliman et al. 2012; Šeda et al. 2017). In one study, a mutation (c.1319G > A; p.Arg440Gln) in *ZBTB16* was identified in brothers with ASD (Bacchelli et al. 2019). We demonstrated that *Zbtb16* KO mice exhibit ASD- and SCZ-like behaviors, including social impairments, repetitive behaviors, risk-taking behaviors, and cognitive deficits, with reduced neocortical thickness, particularly L6 thickness, and reduced numbers of L6 neurons (Fig. 3) (Usui et al. 2021a). In *Zbtb16* KO mice, the numbers of oligodendrocyte progenitors (OPCs) and mature oligodendrocytes were reduced, and myelination of the neocortex was impaired (Fig. 3) (Usui et al. 2021a). The *Zbtb16* transcriptome in the PFC revealed that *Zbtb16* is involved in neurogenesis, nervous system development, cell localization, axon ensheathment, myelination, and the regulation of ASD- and SCZ-associated gene expression (Usui et al. 2021a). In addition, the length of the axonal initial segment of pyramidal neurons was reduced in the primary somatosensory cortex of *Zbtb16* KO mice (Fig. 3) (Usui et al. 2022b), suggesting that the action potential of these neurons may be impaired. These studies demonstrate that L6 and cytoarchitecture, oligodendrocytes, and myelination are essential for the neural basis of sociality.

Abnormalities in L6, such as reductions in the thickness and number of neurons, are common phenotypes in ASD mouse models (Usui et al. 2017a, 2021a; Co et al. 2020a, b). Interestingly, ASD-associated genes have been reported to be enriched in the deep layers (Willsey et al. 2013; Tebbenkamp et al. 2014). In particular, high-confidence ASD and probable ASD risk genes converge to glutamatergic projection neurons in L5 and L6 of the human mid-fetal prefrontal and primary motor-somatosensory cortex (Willsey et al. 2013). Cortical glutamatergic projection neurons can be divided into major classes and subclasses that contribute to distinct functional subnetworks (Harris and Shepherd 2015). The major class consists of pyramidal tract neurons, which give rise to corticofugal pathways that target all subcortical regions (Fig. 3) (Mohan et al. 2023). Another class consists of intratelencephalic neurons that target other cortical and striatal regions, including the contralateral hemisphere (Fig. 3) (Mohan et al. 2023). The L6 pyramidal neurons also project to the claustrum, striatum, and ipsilateral cortex (Fig. 3) (Thomson 2010; Bertero et al. 2022; Baker et al. 2018). Another study demonstrated that the activation of L6 corticothalamic neurons suppresses excitatory neurons in L4 and generates EPSPs in pyramidal neurons in L5a, indicating that L6 corticothalamic neurons strongly activate the output layer of the cortex (Kim et al. 2014). Moreover, the corticothalamic pathways originate from L5 or L6 of the neocortex and are much more numerous than the ascending thalamocortical pathways (Antunes and Malmierca 2021). Interestingly, L6 corticothalamic neurons are also involved in behavioral switching between sound detection and discrimination (Antunes and Malmierca 2021; Guo et al. 2017). Accordingly, L6 neurons coordinate not only auditory information but also various sensory systems, such as visual information, and the integration of this sensory information has been suggested to contribute to the control of social behaviors.

FOXP2 and TBR1 are the L6 markers. TBR1 is a T-box brain transcription factor that was identified in patients with ASD and ID (Nambot et al. 2020; Sapey-Triomphe et al. 2020; Vegas et al. 2018), has been shown to regulate cortical lamina formation, differentiation of L6, dendritic patterning, and inhibitory synaptic density during development (Co et al. 2022; Fazel Darbandi et al. 2018; Bedogni et al. 2010). *Tbr1* haploinsufficiency in mice resulted in axonal projection defects in amygdala neurons and impairments in social interaction, USVs, and cognitive flexibility (Huang et al. 2014). TBR1 chromatin immunoprecipitation sequencing studies have also reported that TBR1-bound regions adjacent to ASD genes are enriched in the developing mouse neocortex (Notwell et al. 2016). *FOXP2* is expressed in deep-layer subcortical projection neurons, such as L6 corticothalamic projection neurons and L5 pyramidal tract neurons (Sorensen et al. 2015; Tasic et al. 2016; Kast et al. 2019; Willsey et al. 2013). *FOXP2* is associated with speech and language disorders, ASD, and attention-deficit/hyperactivity disorder (Lai et al. 2001; Demontis et al. 2019; Reuter et al. 2017; Satterstrom et al. 2020). *Foxp2* cKO mice exhibited decreased USVs, impaired cognitive flexibility, and decreased cortical DRD1 expression (Co et al. 2020a, b). These findings suggest that L6 neurons play essential roles in social behavior, cognition, and output of cortical neuronal activity.

On the other hand, the abnormal laminar cytoarchitecture and cortical disorganization of L2/3 thourt L5 neurons in focal patches in the cortex have been reported in children with ASD (Stoner et al. 2014). This patch phenotype has been reported in the dysgranular zone of the primary somatosensory cortex where showing excessive neuronal activities in maternal immune activation offspring mice model of ASD (Shin Yim et al. 2017). It has also reported that disorganization of cortical networks within L1 in the lateral prefrontal cortex of children with ASD (Trutzer et al. 2019). These reports were observed in the upper layers of the cortex, but excessive neural activity has been reported in not only the upper layers but also the deep layers of the cerebral cortex in mice with the patch phenotype. As our findings related to those, the length of the axonal initial segment of pyramidal neurons was reduced in the L2/3 primary somatosensory cortex of *Zbtb16* KO mice (Usui et al. 2022b). These previous studies show that not only the deep layers, but also upper layers in the cortex.

Taken together, our findings demonstrate that developmental defects in L6 neurons and Purkinje cells which are common in ASD model mice, are responsible for sociality (Co et al. 2020a, b; Usui et al. 2021a, 2017b, 2017a).

## Relationships between oligodendrocytes and sociality

**Fig. 4:**
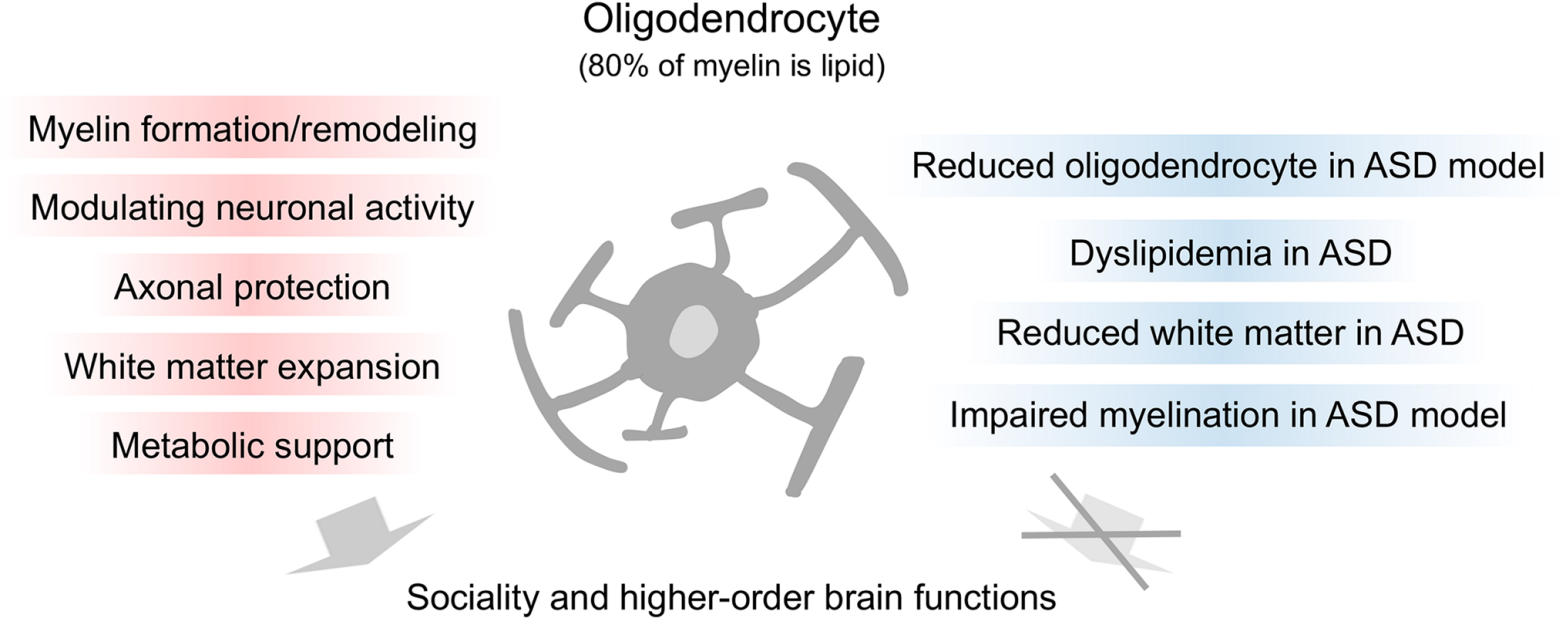
Association between oligodendrocytes and sociability. The red bars indicate the normal function of oligodendrocytes. Oligodendrocytes form myelin and play important roles in modulating neuronal activity, axonal protection, white matter expansion, and maintaining brain functions through metabolism supports. In contrast, the blue bars indicate the associated dysfunction of oligodendrocytes in patients with ASD and in ASD model mice. Dyslipidemia is associated with sociality in children with ASD. Lipids are the major components of myelin, and dyslipidemia in ASD adversely affects myelin structure. Oligodendrocytes are the major components of the white matter, and decreased white matter volume has been reported in patients with ASD, suggesting oligodendrocytes play a role in sociability and other higher order brain functions

Oligodendrocytes are linked to NDDs and psychiatric disorders (Berto et al. 2019; Nagy et al. 2020; Fessel 2022; Zhou et al. 2021; Miyata et al. 2015; Castelijns et al. 2020). In patients with ASD, age-related differences in white matter diffusion have been reported in the uncinate fasciculus, corticospinal tract, inferior longitudinal fasciculus, inferior fronto-occipital fasciculus, anterior thalamic radiation, superior longitudinal fasciculus, and forceps major (Thompson et al. 2020). Studies on ASD severity and white matter development have reported lower white matter development in early childhood than in typical development (Andrews et al. 2021). Consistent with findings in patients with ASD (Barnea-Goraly et al. 2004, AmaralSchumann and Nordahl 2008), impairments in oligodendrogenesis and myelination were found in ASD model mice (Fig. 4) (Usui et al. 2021a). *Tcf4* mutant mice display impaired oligodendrocyte development and myelination, supporting the importance of oligodendrocytes in ASD pathogenesis (Phan et al. 2020). *Chd8* heterozygous mice also exhibit abnormal social behaviors, anxiety-like behaviors, defective myelination, and slower action potential transmission (Kawamura et al. 2020).

Lipids are the primary components of the myelin sheath formed by oligodendrocytes. Dyslipidemia phenotypes in children with ASD have been reported previously (Fig. 4) (Usui et al. 2020), with these children showing positive correlations between clinical scores for social interactions and a specific decrease in very-low-density lipoprotein (VLDL) levels, as well as increases in fatty acid levels (Usui et al. 2020). Furthermore, the activity of lipoprotein lipase, a VLDL-degrading enzyme, is higher in children with ASD (Hirai et al. 2020), suggesting that VLDL is specifically degraded during ASD pathogenesis. Interestingly, VLDL and low-density lipoprotein receptors are specifically expressed in mature myelinating oligodendrocytes at postnatal stages but are progressively downregulated after P15 (Zhao et al. 2007), suggesting that VLDL and low-density lipoprotein play important roles in myelination. These studies suggest that oligodendrocytes contribute to higher order brain functions in humans, while oligodendrocyte impairment is a risk factor for disorders such as NDDs and cognitive disorders and plays an essential role in sociality (Fig. 4).

## Conclusion

This review focused primarily on studies aimed at understanding the neural basis of sociality. Studies have also characterized the molecular mechanisms by which sociality is acquired by focusing on human brain evolution. Moreover, focusing on brain disorders that impair sociality like ASD as a disorder characterized by social impairment, studies have demonstrated many sociability-related brain phenotypes. A combination of these two approaches may uncover the neural circuits and human-specific gene expression networks that are suggested to be related to sociality. From these methods, we suggested that layer 6 of the cerebral cortex and oligodendrocytes are related to the neural basis of human sociality.

However, unlike specific genes, it is difficult to actually study the complete function of the gene networks themselves identified from studies focused on brain evolution. Therefore, it is possible to analyze the function of highly important genes such as “hub genes” on the network instead of expressing the genes network themselves. In order to elucidate the functions of human genes, studies that expresses human genes in model animals has provided many findings (Geschwind and Konopka 2012; Charrier et al. 2012; Dennis et al. 2012). On the other hand, it is also true that model animals have the problem that they do not have the same genomic background as humans.

To avoid such problems, there are methods using human cells or human brain organoids (Doi et al. 2022a, b; Gordon and Geschwind 2020). Similarly, to elucidate the function of human oligodendrocytes, there are options to use cultured human oligodendrocytes, cultured brain organoids, or even engraftment of human oligodendrocytes in the model animal brain such as mouse to analyze their effects at the individual level. We will elucidate the function of these human hub genes and human oligodendrocytes in the future studies. Particularly, we are particularly interested in determining how the capabilities and functions of human oligodendrocytes differ from those of other primates and rodents. The latest study shows that OPCs are specifically increased in human posterior cingulate cortical tissue, while mature oligodendrocytes are decreased, suggesting OPCs accelerated human brain evolution (Caglayan et al. 2023). Moreover, we will investigate the involvement of oligodendrocytes and their myelination in the development and regulation of sociality, which is relevant to the pathogenesis of ASD and human brain evolution.

In addition, it is important to elucidate how neural circuits centered on cortical L6 control sociality, a common phenotype observed in several ASD mouse models. In the L6 of the cerebral cortex, genes related to ASD and sociality, and genes such as *FOXP2* related to language are expressed, indicating functional importance for sociality. Interestingly, the latest study also shows *FOXP2* expression is specifically increased in two excitatory subtypes (L4-6_RORB_2 and L5-6_THEMIS_1 subtypes) of posterior cingulate cortex, compared to other primates (Caglayan et al. 2023). Therefore, we will focus on the output from L6 and input to L6 in the cerebral cortex, and will investigate the neural circuits related to sociality including the interaction with oligodendrocytes using transgenic animals, optogenetics, and chemogenetics.

In close, we will continue to study these topics to elucidate a complete picture of the neural basis underlying human sociality for providing the insight into the neural basis of sociality.
